# An operational approach for estimating pulmonary diffusing capacity for oxygen in humans

**DOI:** 10.1113/EP093578

**Published:** 2026-02-24

**Authors:** Stine B. Nymand, Rie Skovly Thomsen, Iben Elmerdahl Rasmussen, Ronan M. G. Berg

**Affiliations:** ^1^ Centre for Physical Activity Research Copenhagen University Hospital – Rigshospitalet Copenhagen Denmark; ^2^ Department of Biomedical Sciences, Faculty of Health and Medical Sciences University of Copenhagen Copenhagen Denmark; ^3^ Department of Clinical Physiology and Nuclear Medicine Copenhagen University Hospital – Rigshospitalet Copenhagen Denmark; ^4^ Department of Clinical Medicine, Faculty of Health and Medical Sciences University of Copenhagen Copenhagen Denmark; ^5^ Neurovascular Research Laboratory, Faculty of Life Sciences and Education University of South Wales Pontypridd UK

The measurement of pulmonary diffusing capacity (*D*_L_), also known as transfer factor (*T*_L_), is used to assess lung function in both mechanistic physiological studies, for example on the pulmonary adaptations and limitations to exercise and/or postural changes (Madsen et al., 2023; Tedjasaputra et al., 2016; Thomsen, Rasmussen, Nymand, et al., 2025; Zavorsky & Smoliga, 2017) and in in clinical practice, where disease‐specific reductions are well‐known across obstructive, restrictive and vascular lung diseases (Hughes & Pride, 2012). While this typically involves the use of carbon monoxide (CO) or nitric oxide (NO), which are reported and interpreted in their own right, these test gases should, for the most part, be regarded as proxies for evaluating the diffusive transport of the respiratory gases, particularly O_2_ but also CO_2_ (Hughes & Borland, 2015). Accordingly, when Marie Krogh (1874–1943) developed the single‐breath technique and used CO as the test gas more than a century ago (Berg, 2024), she was in fact attempting to investigate O_2_ transfer, assuming an O_2_–CO diffusivity ratio of 1.23, as based on their respective molecular weights and solubilities in the alveolar–capillary membrane (Krogh, 1915). However, she was unaware that a substantial proportion of the diffusive resistance to both gases, to different extents, resides within the red blood cell (Hughes & Bates, 2003). This only became clear years later when Francis J. W. Roughton (1899–1972) and Robert E. Forster (1914–2006) derived their famous equation (Roughton & Forster, 1957), described here for a given gas *x*:

$$\frac{1}{D_{L,x}}=\frac{1}{D_{M,x}}+\frac{1}{D_{B,x}}$$

where *D*_M,_ *_x_* denotes the alveolar–capillary conductance (including the plasma layer between the endothelium and the red blood cell extracellular membrane) for *x*, and *D*_B,_ *_x_* the conductance for the gas into red blood cells in the pulmonary capillary bed. Of note, D_B,_ *_x_* is the product of pulmonary capillary blood volume, *V*_c_, and the specific blood conductance, θ*_x_*, of which the latter is a composite of the conductance of the extracellular membrane and intracellular fluid of the red blood cell for *x* and its rate of chemical combination with haemoglobin. The Roughton–Forster equation can be solved experimentally to provide *D*_M,CO_ and *V*_C_​ either by measuring *D*_L,CO_ at different alveolar $P_{O_{2}}$ levels or by using a single manoeuvre with to obtain a simultaneous measurement of pulmonary CO and NO uptake (Borland & Hughes, 2020; Zavorsky et al., 2017).

The abovementioned considerations bring us back to the main measure of (physiological) interest here: $D_{L,O_{2}}$. This metric has been measured directly in previous studies, but the existing procedures are exceedingly difficult (Hammond & Hempleman, 1987; Hyde et al., 1966; Lilienthal et al., 1946; Siebens et al., 1959). However, given that Krogh's O_2_–CO diffusivity ratio of 1.23 applies to *D*_M_, determining *D*_M,CO_, together with *V*_c_, does allow for the calculation of the $D_{L,O_{2}}$, provided that $\theta_{O_{2}}$ is known. But this is by no means straightforward (Hsia et al., 2016): $\theta_{O_{2}}$ is not constant in vivo; it depends on red blood cell size, as well as the O_2_ saturation of haemoglobin and haemoglobin concentration. Differences in red blood cell size are primarily relevant for between‐species comparisons and will not be considered further here. However, within humans, the dominant source of variation arises from changes in haemoglobin saturation as blood traverses the pulmonary capillary bed.

By studying thin films of whole blood directly exposed to air, Heidelberger and Reeves demonstrated how whole‐blood $\theta_{O_{2}}$ varies with haemoglobin saturation (Heidelberger & Reeves, 1990): it is low at very low saturation, increases to a maximum at intermediate saturation (approximately 0.40–0.50), and then declines steeply as saturation approaches unity. When these data are used to perform a Bohr integration, assuming a mixed venous saturation of approximately 0.65 and an end‐capillary saturation near 0.98, the effective $\theta_{O_{2}}$ in the pulmonary capillary becomes ∼0.6 mmol mL^−1^ min^−1^ kPa^−1^, in agreement with a previously recommended standard value in humans (Hsia et al., 2016). Importantly, according to the data by Heidelberger and Reeves, changes in mixed venous saturation alone will have little influence on the effective $\theta_{O_{2}}$ provided that end‐capillary saturation still approaches unity. Thus, even during strenuous exercise, where mixed venous saturation may fall to ∼0.40 (Mortensen et al., 2008; Wright et al., 2016), the effective $\theta_{O_{2}}$ will remain close to its resting value because the Bohr integration is dominated by the low‐conductance, high‐saturation region near the end of the capillary. In contrast, conditions that reduce end‐capillary saturation will exert a much larger influence on the effective $\theta_{O_{2}}$. Thus, severe alveolar hypoxia, critically reduced red blood cell transit time through the pulmonary capillary, or an adverse interaction between the two, as occurs with marked ventilation–perfusion mismatch, will limit the rise in saturation toward unity and thus shift the integration toward the intermediate saturation range where $\theta_{O_{2}}$ is maximal. For example, if saturation increases only from approximately 0.53 to 0.85 between mixed venous and end‐capillary blood, $\theta_{O_{2}}$ will be in the order of 1.3–1.5 mmol O_2_ mL^−1^ min^−1^ kPa^−1^. These considerations indicate that while assuming a constant $\theta_{O_{2}}$ is reasonable under most physiological conditions, with 0.6 mmol O_2_ mL^−1^ min^−1^ kPa^−1^ being the best current estimate, caution is warranted in the presence of arterial hypoxaemia arising from these mechanisms.

With respect to haemoglobin concentration, whole‐blood $\theta_{O_{2}}$ scales approximately in proportion to haemoglobin. Variation in haemoglobin can therefore be accounted for by a simple linear correction (Hsia et al., 2016):

$$\;\mathrm{Adjusted}\;\theta_{O_{2}}=\theta_{O_{2}}\;\;\frac{\left[\mathrm{Hb}\right]}{9.3\;\mathrm{mM}}$$

In support of assuming a constant $\theta_{O_{2}}$ across various experimental settings including both exercise and lung disease, this has previously been compared to measurements by the multiple inert gas elimination technique, the gold standard for pulmonary gas exchange measurements, in dogs across states of both normal and reduced lung function, and been shown to validly predict diffusive O_2_ transfer from rest to heavy exercise (Hsia et al., 2008).

By assuming a $\theta_{O_{2}}$ of 0.6 mmol mL^−1^ min^−1^ kPa^−1^, the following operational equation can be obtained to derive an estimated $D_{L,O_{2}}$ ($eD_{L,O_{2}}$; see also Appendix 1):

$$eD_{L,O_{2}}=\frac{0.738\;\cdot \;D_{M,\mathrm{CO}}\;\cdot \;V_{C}}{0.6\;\cdot \;V_{C}+1.23\;\cdot \;D_{M,\mathrm{CO}}}$$

In Table 1, we provide $eD_{L,O_{2}}$ as obtained in healthy young volunteers (mean age 25 [SD 1.5] years, male–female ratio 1:1) from a previous study, in which *D*_L,CO_ and *D*_L,NO_ were simultaneously determined during upright rest, in the supine posture and during exercise at an intensity corresponding to 90% of the ventilatory threshold (Madsen et al., 2023).

**TABLE 1:** Estimated pulmonary diffusing capacity for oxygen in healthy volunteers.

	Upright rest	Supine	Exercise
*n*	20	9	11
$eD_{L,O_{2}}$ (mmol min^−1^ kPa^−1^)	21.8 (4.2)	22.5 (4.5)	29.4 (5.4)
SRD (mmol min^−1^ kPa^−1^)	2.7 [2.0, 4.1]	2.4 [1.9, 3.2]	2.5 [1.7, 4.0]
CV (%)	4.2 [2.9, 5.5]	3.3 [1.8, 4.8]	2.7 [1.6, 3.7]

*Notes*: Data are presented as mean (SD) and with 95% CI [LL, UL] for the reliability indices. Measurements were obtained from a previous study (Madsen et al., 2023), in which the dual test gas diffusing capacity for carbon monoxide and nitric oxide was measured twice in healthy young volunteers 2–10 days apart. Data from study Day 1 and 2 have been pooled in the analysis (upright rest: *n* = 2 × 20; supine: *n* = 2 × 9; exercise, *n* = 2 × 11).

Abbreviations: CV, coefficient of variation; $eD_{L,O_{2}}$, estimated pulmonary diffusing capacity for oxygen; SRD, smallest real difference.

Inspired by concepts such as clinimetrics and psychometrics, ‘physiolometrics’ has recently been proposed as a framework for the advancement and evaluation of physiological measurement methods (Hartmann et al., 2023), including the systematic assessment of their validity and reliability. Thus, in terms of logical validity, this approach rests on the Roughton–Forster equation, albeit with the inherent limitations mentioned above, which must be recognised and taken into account when reporting $eD_{L,O_{2}}$. As for construct validity, previously reported measurements at rest in healthy individuals based on other methods have provided lower values for $D_{L,O_{2}}$ than reported here, ranging from 7 (mean, SD 3) mmol min^−^^1^ kPa^−^^1^ and 8–9 mmol min^−^^1^ kPa^−^^1^ as based on invasive catheterisation (Lilienthal et al., 1946; Turino et al., 1963), to 11 (mean, SD 3) mmol min^−^^1^ kPa^−^^1^ based on the pulmonary uptake of ^34^O_2_ (Hyde et al., 1966) and 14 (mean, SD 2) mmol min^−^^1^ kPa^−^^1^ using the multiple inert gas elimination technique (Hammond & Hempleman, 1987). However, in one study based on the pulmonary uptake of ^18^O_2_ and another where a Roughton–Forster equation‐based approach was also used, mean values of 18 (SD 3) and of 19 (SD 2) mmol min^−^^1^ kPa^−^^1^, respectively, were reported (Hyde et al., 1967; Meyer et al., 1981). These are clearly closer to the current estimates; the reason why the mean Roughton–Forster equation‐based mean value is slightly lower than here is probably that the estimates were based on a lower $\theta_{O_{2}}$ (Staub et al., 1962). In terms of the data provided here (Table 1), the observed increases in $eD_{L,O_{2}}$ from upright rest in the supine posture (mean 1.9 [95% CI: 1.2, 2.6] mmol min^−^^1^ kPa^−^^1^, *P* = 0.0004) and during exercise (mean 6.8 [95% CI: 6.0, 7.6] mmol min^−^^1^ kPa^−^^1^, *P* < 0.00001) are also consistent with findings in previous studies (Hammond & Hempleman, 1987; Hsia et al., 2008; Meyer et al., 1981; Turino et al., 1963). Together, these findings thus support the construct validity of the outlined approach for deriving $eD_{L,O_{2}}$.

Given that the measurements provided in Table 1 were repeated in an identical experimental set‐up on two separate days, the corresponding between‐day test–retest reliability indices for each condition are also provided. The smallest real difference (SRD) is a measure of absolute reliability that estimates the maximal difference, in the given units of measure, between two measurements on 95% of occasions when no true underlying difference is present, thereby providing the minimal difference that can be interpreted as real and exceeding the expected measurement error. The coefficient of variation (CV) is a measure of relative reliability that expresses the proportion of variance attributable to measurement error. Both reliability indices are provided for $eD_{L,O_{2}}$ in Table 1 for future reference. A cutoff for CV is difficult to define, but there is some consensus that a value below 10% is an acceptable threshold for physiological measurement methods (Mohammad et al., 2025). As shown in Table 1, SRD for $eD_{L,O_{2}}$ is similar across conditions, ranging from 2.4 to 2.7 mmol min^−^^1^ kPa^−^^1^, with a CV consistently below 10%.

Because measurements that allow derivation of *D*_M,CO_ and *V*_C_ are not widely available in the clinical setting, and keeping in mind that *D*_L,CO_ itself is widely used to diagnose, classify and monitor lung disease and treatment effects, it is unlikely that $eD_{L,O_{2}}$ will find any direct clinical application in the very near future. Nevertheless, it is well documented that different lung diseases affect *D*_M,CO_ and *V*_C_ in distinct ways, often with substantial interindividual variability even within the same disease category (see for example, Barisione et al., 2019; Farha et al., 2013; Lytzen et al., 2024; Magini et al., 2021; Thomsen, Mohammad, Ragborg, et al., 2025). From a physiological and potentially clinical perspective, the most relevant consequence of such disease‐specific alterations may indeed be their combined impact on $D_{L,O_{2}}$, and it is worth exploring whether the operational approach outlined here can help to clarify these relationships, while fully acknowledging the inherent assumptions and limitations described above. A logical first step would be the development of empirical regression equations to derive reference values for estimated $eD_{L,O_{2}}$ in healthy individuals based on sex, age and height, analogous to existing reference material for *D*_M,CO_ and *V*_C_ (Munkholm et al., 2018). In any event, in mechanistic studies addressing various aspects of O_2_ uptake and lung physiology, the operational equation for $eD_{L,O_{2}}$ provided here seems to offer valid and reliable measures that are physiologically meaningful and intuitive to interpret.

## AUTHOR CONTRIBUTIONS

Stine B. Nymand: Data collection, analysis and interpretation, table and revisions. Rie Skovly Thomsen: Data collection, data interpretation and revisions. Iben E. Rasmussen: Data collection, data interpretation and revisions. Ronan M. G. Berg: Conception, data interpretation, first draft, table, revisions and supervision. All approved the final version of the manuscript and agree to be accountable for all aspects of the work in ensuring that questions related to the accuracy or integrity of any part of the work are appropriately investigated and resolved. All persons designated as authors qualify for authorship, and all those who qualify for authorship are listed.

## CONFLICT OF INTEREST

None declared.

## APPENDIX 1 DERIVING AN OPERATIONAL EQUATION FOR ESTIMATING THE PULMONARY DIFFUSING CAPACITY FOR OXYGEN ($D_{L,O_{2}}$)

The Roughton–Forster equation states that:

$$\frac{1}{D_{L,O_{2}}}=\frac{1}{D_{M,O_{2}}}+\frac{1}{V_{c}\cdot \;\theta_{O_{2}}}$$

Given that the O_2_–CO diffusivity ratio is 1.23 (see below), then:

$$\frac{1}{D_{L,O_{2}}}=\frac{1}{1.23\cdot D_{M,\mathrm{CO}}}+\frac{1}{V_{c}\cdot \;\theta_{O_{2}}}$$

If $\theta_{O_{2}}$ is assumed to be 0.6 mmol mL^−1^ min^−1^ kPa^−1^, then an estimated $D_{L,O_{2}}$ ($eD_{L,O_{2}}$) can be derived:

$$\begin{array}{ccc} \;eD_{L,O_{2}} & & =\frac{1.23\cdot 0.6\;\cdot \;D_{M,\mathrm{CO}}\cdot \;V_{C}}{0.6\cdot V_{C}+1.23\cdot D_{M,\mathrm{CO}}}\\ & & =\frac{0.738\;\cdot \;D_{M,\mathrm{CO}}\cdot \;V_{C}}{0.6\cdot V_{C}+1.23\cdot D_{M,\mathrm{CO}}}\left(\mathrm{mmol}\;\mathrm{mi}n^{-1}\;\mathrm{kP}a^{-1}\right) \end{array}$$

If $eD_{L,O_{2}}$ is instead reported in mL^−^^1^ min^−^^1^ mmHg^−^^1^, then $\theta_{O_{2}}$ is 1.8 mL O_2_ mL^−1^ min^−^^1^ mmHg^−^^1^, and the equation becomes:

$$\begin{array}{ccc} eD_{L,O_{2}} & & =\frac{1.23\cdot 1.8\cdot D_{M,\mathrm{CO}}\cdot V_{C}}{1.8\cdot V_{C}+1.23\cdot D_{M,\mathrm{CO}}}\\ & & =\frac{2.214\cdot D_{M,\mathrm{CO}}\cdot V_{c}}{1.8\cdot V_{c}+1.23\cdot D_{M,\mathrm{CO}}}\left(\mathrm{mL}\;\mathrm{mi}n^{-1}\;\mathrm{mmH}g^{-1}\right) \end{array}$$

The O_2_–CO diffusivity ratio is theoretically derived from the Krogh formulation (Krogh, 1915), in which the diffusivity of a gas through the (water‐like) alveolar–capillary membrane is proportional to its solubility coefficient and inversely proportional to the square root of its molecular weight. Accordingly, the ratio depends only on the relative solubilities of O_2_ and CO and their molecular weights and is independent of physiological or metabolic state. While gas solubility in water decreases modestly with increasing temperature, the temperature dependencies of O_2_ and CO solubility are closely matched. Based on recommended Henry's law constants for and their temperature dependence for aqueous solutions (Sander, 2023), the resulting variation in the diffusivity ratio is less than 1% between 36°C and 41°C and the impact of temperature is therefore physiologically negligible. The ratio is thus treated as constant for operational purposes.
